# Open modeling and exchange (OMEX) metadata specification version 1.0

**DOI:** 10.1515/jib-2020-0020

**Published:** 2020-06-25

**Authors:** Maxwell L. Neal, John H. Gennari, Dagmar Waltemath, David P. Nickerson, Matthias König

**Affiliations:** Seattle Children’s Research Institute, Seattle, USA; University of Washington, Seattle, USA; University Medicine Greifswald, Greifswald, Germany; University of Auckland, Auckland, New Zealand; Humboldt University Berlin, Berlin, Germany

**Keywords:** data integration, metadata, semantics, simulation

## Abstract

A standardized approach to annotating computational biomedical models and their associated files can facilitate model reuse and reproducibility among research groups, enhance search and retrieval of models and data, and enable semantic comparisons between models. Motivated by these potential benefits and guided by consensus across the COmputational Modeling in BIology NEtwork (COMBINE) community, we have developed a specification for encoding annotations in Open Modeling and EXchange (OMEX)-formatted archives. Distributing modeling projects within these archives is a best practice established by COMBINE, and the OMEX metadata specification presented here provides a harmonized, community-driven approach for annotating a variety of standardized model and data representation formats within an archive. The specification primarily includes technical guidelines for encoding archive metadata, so that software tools can more easily utilize and exchange it, thereby spurring broad advancements in model reuse, discovery, and semantic analyses.

## OMEX Metadata Specification version 1.0

April 15, 2020

**Lead Developer:** Maxwell Neal

**Editors**

| | |
|---|---|
| John Gennari | *University of Washington* |
| Dagmar Waltemath | *Universitätsmedizin Greifswald* |
| David Nickerson | *University of Auckland* |
| Matthias König | *Humboldt-Universität zu Berlin* |

The latest release of the specification is available at:
https://co.mbine.org/specifications/omex-metadata

To discuss any aspect of the current specification as well as language details, please post a message to the COMBINE annotation forum:
https://groups.google.com/forum/#!forum/combine-annot

## Contents

1 Introduction 4
1.1 Motivation . . . . . 4
1.1.1 Cross-format search . . . . . 4
1.1.2 Semantic similarity between models . . . . . 4
1.1.3 Semantics-based composition . . . . . 4
1.1.4 Semantic integration of empirical data and simulation models . . . . . 4

2 OMEX Metadata technical specification 5
2.1 Conventions used in this document . . . . . 5
2.2 Concepts used in OMEX Metadata . . . . . 5
2.2.1 COMBINE archives . . . . . 5
2.2.2 RDF . . . . . 5
2.2.3 Model-level annotations . . . . . 5
2.2.4 Semantic annotations . . . . . 5
2.2.5 Non-semantic annotations . . . . . 6
2.2.6 Singular annotations . . . . . 6
2.2.7 Composite annotations . . . . . 6
2.2.8 identifiers.org URIs . . . . . 6
2.2.9 BioModels.net qualifiers . . . . . 6
2.2.10 Metadata identifiers . . . . . 6
2.3 Serializing OMEX Metadata . . . . . 6
2.3.1 Serialization format . . . . . 7
2.3.2 Separation of annotations from models and data . . . . . 7
2.3.3 Formatting URIs in RDF . . . . . 7
2.3.4 Serializing non-semantic annotations . . . . . 8
2.3.5 Serializing singular annotations . . . . . 8
2.3.6 Serializing composite semantic annotations . . . . . 9
2.3.7 Annotating tabular data . . . . . 12
2.3.8 Annotating physical units . . . . . 12
2.4 Knowledge resources to use for annotation . . . . . 13
2.4.1 Resources to use for model-level annotations . . . . . 13
2.4.2 Resources to use for composite semantic annotations . . . . . 13
2.4.3 Resources to use for singular physical property annotations . . . . . 13
2.4.4 Internally referencing biological knowledge stored within COMBINE archives . . . . . 14
2.5 OMEX Metadata resources . . . . . 14

3 Acknowledgements 15

A OMEX Metadata examples 15

B The COMBINE archive 15

## 1 Introduction

### 1.1 Motivation

Metadata annotations enhance the interoperability, reusability, comparability, and comprehension of computational biological models. Annotations can capture the biological meaning of what a model simulates, specify precisely the components comprising a model, describe a model's provenance, provide layout information for visualizing a model's architecture, etc. These annotations can be leveraged to make it easier for researchers to find and re-purpose models, re-combine models and model parts, and integrate models across repositories and experimental data stores. For example, semantic annotations can be leveraged to enhance model search capabilities by identifying models that overlap in terms of the biological phenomena they represent.

Realizing the potential benefits of annotation requires the development of standards that adhere to a community-based annotation protocol. Without such standards, researchers must account for a variety of annotation formats and approaches, a situation that can become prohibitively cumbersome and which can defeat the purpose of annotating a model.

This document was created to specify how to represent model annotations within the Open Modeling and EXchange (OMEX) file format [1]. Our goal is to harmonize and simplify the representation of metadata annotations in models that are shared among the biological research community regardless of the models' encoding format. Our hope is that community-wide adherence to this specification will significantly advance the community's ability to discover relevant models and data sets as well as to re-purpose/re-combine models and model components.

#### 1.1.1 Cross-format search

Researchers cannot easily search across model repositories for models that simulate a particular biological process. As illustrated by Henkel, et al. [2], if the annotations on models in various repositories were encoded according to a common standard, this would make it easier to develop tools for searching across repositories and modeling formats.

#### 1.1.2 Semantic similarity between models

Using standardized metadata annotations to capture the biological properties simulated by a model allows developers to quantify how similar two models are in terms of the biological phenomena they represent (see, for example, [3, 4]). Such objective measures of biological similarity are critical for developing tools that help users discover related models within and across model repositories, exposing users to new models that may be relevant to their research.

#### 1.1.3 Semantics-based composition

Thorough semantic annotations on models are also critical for performing semantics-based model composition. This compositional approach, which aims to reduce the time and code-level edits required to merge models into larger systems, leverages machine-readable semantic annotations to automatically propose biologically-consistent interfaces between models [5]. The use of a consistent annotation protocol is necessary for achieving this level of composability for biological models.

#### 1.1.4 Semantic integration of empirical data and simulation models

Given that models are largely intended to reproduce, explain, and predict empirical data measurements, any general solution for annotating model elements would also be applicable for annotating empirical data used for model parameterization and validation. For example, the same semantic annotation on a CellML model variable that represents aortic blood pressure could be used to annotate an empirical aortic blood pressure measurement recorded in a data file. Using a common, standardized approach for annotating models as well as empirical data would accelerate the development of tools that help modelers discover data sets of interest for model parameterization or validation and help experimentalists discover models of interest for use in analyses.

## 2 OMEX Metadata technical specification

This section presents the technical specification for associating metadata with the contents of COMBINE archives [1] in the OMEX file format.

### 2.1 Conventions used in this document

Resource Description Framework (RDF, https://www.w3.org/RDF/) content and in-paragraph references to RDF subjects, predicates, and objects are 1 indicated by `typewriter font`.

The table below indicates the prefixes used in RDF Uniform Resource Identifiers (URIs) and their namespaces.

| Prefix | Namespace |
|---|---|
| rdf | http://www.w3.org/1999/02/22-rdf-syntax-ns# |
| dcterms | http://purl.org/dc/terms/ |
| bqbiol | http://biomodels.net/biology-qualifiers/ |
| bqmodel | http://biomodels.net/model-qualifiers/ |
| semsim | http://www.bhi.washington.edu/semsim# |

### 2.2 Concepts used in OMEX Metadata

#### 2.2.1 COMBINE archives

A COMBINE archive (also known as an OMEX archive) is a single file containing the various documents necessary for the description of a model as well as all associated data and simulation procedures. These documents include, for example, simulation experiment descriptions, all models needed to run the simulations, associated data files, etc. The archive is encoded using the OMEX format. Version 1 of the OMEX specification is available at https://co.mbine.org/freelinking/standards/omex/version-1.

#### 2.2.2 RDF

The Resource Description Framework (RDF) is a World Wide Web Consortium-recommended standard for representing information on the Web. RDF consists of statements built using subject-predicate-object triples that can be used to assert relationships between model components and terms from online knowledge resources. A primer on RDF is available at https://www.w3.org/TR/rdf11-concepts/, and links to examples of RDF-encoded model annotations can be found in this document.

#### 2.2.3 Model-level annotations

A model-level annotation is an annotation that captures an aspect of the model *as a whole*. Examples include an annotation that indicates the PubMed ID of the model's source publication, an annotation that indicates that the model simulates glycolysis, or an annotation that indicates the identity of the curator who encoded the model.

#### 2.2.4 Semantic annotations

A semantic annotation is a computer-accessible metadata item that captures, entirely or in part, the meaning of a model, model component or data element. For example, an annotation on a model variable might indicate that it represents the concentration of cytosolic glucose in a pancreatic beta cell or it might describe a purely computational feature such as the simulation time step. To further clarify, we make a distinction between semantic annotations and other types of metadata that do not indicate meaning, such as curatorial annotations that indicate model authorship, provenance annotations that indicate the origins of model components (e.g., nanopublications) or tool-dependent annotations that indicate layout information for model visualization.

#### 2.2.5 Non-semantic annotations

Non-semantic annotations are metadata items that capture information other than the meaning of the annotated element. Curatorial annotations, provenance and layout annotations are examples of non-semantic annotations.

#### 2.2.6 Singular annotations

Singular annotations are those that are comprised of a single RDF statement linking a model or data element to a knowledge resource term. These are the types of annotations currently found throughout curated models on BioModels. See section 2.3.5 for examples.

#### 2.2.7 Composite annotations

Composite annotations are semantic annotations that are comprised of multiple annotation terms linked using standard qualifiers (also known as "relations" or "predicates") to indicate the precise biological meaning of a model or data element. Composite annotations are used when a single knowledge resource term is not available to define a model or data element. Composite annotations have two primary components: the physical property represented by the annotated item (e.g., chemical concentration, fluid volume) and the physical entity, process, force, or dependency that bears the property (e.g., a pool of ATP, blood in a cardiac cavity, the glucokinase reaction). See section 2.3.6 for examples.

#### 2.2.8 identifiers.org URIs identifiers.org [6] is a resolving system that enables referencing of data for the scientific community with a focus on the life sciences domain. It handles persistent identifiers in the form of URIs and Compact URIs (CURIEs). identifiers.org also provides standardized URI prefixes for a large set of biological knowledge resources.

#### 2.2.9 BioModels.net qualifiers

BioModels.net qualifiers are a set of standardized relations (also known as "predicates") used to indicate the nature of the relationship articulated in an annotation, or in an annotation component. For example, the BioModels.net qualifier `is` is used to indicate the identity of an annotated element and the qualifier `isEncodedBy` is used to indicate that a particular protein is encoded by a particular DNA sequence.

#### 2.2.10 Metadata identifiers

In standardized XML-based model exchange formats such as the Systems Biology Markup Language (SBML [7]), CellML [8], NeuroML [9] and the Simulation Experiment Description Markup Language (SED-ML [10]), the XML elements often have an attribute for specifying a metadata identifier (ID). For example, the following SBML code from model BIOMD0000000001 on BioModels.net indicates that the model has metadata ID "_000001".

```
<model metaid="_000001" id="BIOMD0000000001" name="Edelstein1996 - EPSP ACh event">
...
</model>
```

These metadata IDs are unique to each XML element and are used in annotation statements to link annotations to the XML elements in a COMBINE archive that they describe.

### 2.3 Serializing OMEX Metadata

The specification for serializing annotations in OMEX-formatted documents is based largely on the article by Neal et al. [11], which presents a list of recommendations for standardizing semantic annotations on biological models. This part of the specification addresses how to standardize the storage of annotations within COMBINE archives, an essential technical prerequisite for harmonizing their representation across model annotation efforts.

#### 2.3.1 Serialization format

We recommend encoding OMEX metadata in RDF. RDF has emerged as the *de facto* standard for encoding annotations among the COMBINE community, and all COMBINE standards currently use it. Although more expressive knowledge representation formats exist, RDF is sufficiently expressive for articulating the kinds of annotations required to catalyze significant advances in model discovery, reuse and integration. We recommend RDF content be formatted as RDF/XML as this is the format most widely supported by software libraries. However, annotations can be formatted as Turtle (https://www.w3.org/TR/turtle/) or *n*-triples (`https://www.w3.org/TR/n-triples/`) as well. Software that supports reading/writing COMBINE archive annotation files should support these alternative formats in addition to RDF/XML.

#### 2.3.2 Separation of annotations from models and data

We recommend using separate RDF files to store all annotations associated with model and simulation protocol files within a COMBINE archive. The traditional practice within the COMBINE community has been to serialize annotations within the same file that specifies the model's computational aspects. There are several reasons why we recommend storing semantic annotations in a separate file, instead. First, this will normalize the format in which annotations are stored across the different COMBINE standards. Currently, the exact format used to store annotations within model files differs among standards. Normalizing the format will simplify the development of software that provides programmatic manipulation of annotations. It will also allow for better separation between modeling and annotation tasks, removing the burden of supporting annotation from the software teams that are developing software libraries for specific COMBINE standards.

We also recommend storing annotation files separately because we recognize that different research groups may have different preferences for which knowledge resources to use for annotation. Externalizing annotations in a separate file allows a single model file to be referenced by multiple annotation files, allowing different research groups to describe the same modeling resource in different ways. This approach follows the vision of the COMBINE archive, wherein multiple types of modeling files are archived together to make simulation experiments readily reproducible and shareable among research groups. When sharing models, we recommend that annotations be distributed along with the files they annotate, and COMBINE archives provide a standardized way to bundle such files together. An additional advantage of storing annotations in a separate file is that the RDF content can be serialized in various formats, including XML or Turtle. Currently, the serialization is dictated by the model format.

Storing annotations in a separate file requires keeping them synchronized. For example, if a variable identifier changes in the model file, that change should be reflected in the annotation file(s) as well. We recommend that the community encourages the development of software libraries and tools that help ensure coordination between a model's computational aspects and its annotations.

Multiple RDF annotation files are allowed within an archive and the OMEX manifest file should provide sufficient information so that parsers can automatically determine which files within an archive contain the RDF annotations. Software tools should provide support for reading the content of each individual annotation file into separate RDF graphs and, alternatively, for reading the content of multiple files into one merged RDF graph.

#### 2.3.3 Formatting URIs in RDF

The subjects of RDF triples used for annotation should be relative URIs, giving the name of the file to be annotated, and the metadata ID of the annotated element within the file as the URI fragment. To emphasize that the URI is local to the archive it may be prepended with "./", although this is not required. For example, if there is a model file in a COMBINE archive named MyModel.xml, and it contains an element with metadata ID "meta0", then the subject URI used in an annotation statement on that model element would be:

```
./MyModel.xml#meta0
```

Wherever possible, COMBINE archive annotation documents should use BioModels.net qualifiers in RDF statements that define model elements. These existing qualifiers provide a basic level of coverage needed for articulating annotations in models, and they are specifically intended for use in statements that link computational abstractions of physical phenomena to knowledge resource terms representing the material manifestations of those phenomena.

In addition to BioModels.net qualifiers to encode singular annotations, SemSim qualifiers should be used in composite annotations to unambiguously encode the relationships between the annotation's components (see 2.3.6). As illustrated in the examples in 2.3.6, SemSim qualifiers are primarily used to indicate physical entity participation in a physical process or physical force as well as the stoichiometry of a process's participants.

We recommend using the identifiers.org URI format when referencing knowledge resource terms in RDF statements: identifiers.org supports a vast set of biological knowledge resources used for annotation and identifiers.org-formatted URIs are resolvable. The identifiers.org services are also capable of more complex URI resolution compared to alternative services. For example, identifiers.org is specifically built to address downtime and changing endpoints and directs users to an alternative site for a given data record as long as one is listed (one-to-many mappings) whereas persistent uniform resource locator services specify only one endpoint for URI resolution (one-to-one mappings). We also recommend using identifiers.org-formatted URIs because they use a simple, uniform nested structure that facilitates generation and parsing, and because identifiers.org reuses data providers' record identifiers.

#### 2.3.4 Serializing non-semantic annotations

While the COMBINE community has spent considerable time debating how to standardize semantic annotations, more discussion is needed on how to standardize other types of annotations such as those that describe model curation, provenance (including nanopublications), confidence levels, or layout information for model visualization tools. We therefore recommend that the community identifies the minimal requirements for non-semantic annotations, comes to a consensus about how to serialize them, and then revises this specification document once consensus is achieved. We note that it is important that any future additions to this specification document are in agreement with the COMBINE archive specification document. We also note that there is currently a proposal to use ORCID identifiers rather than VCard-formatted RDF blocks for indicating the identities of people in these types of non-semantic annotations. We encourage further debate on this issue within the community.

#### 2.3.5 Serializing singular annotations

Singular annotations within COMBINE archive annotation files should be encoded as a single RDF triple. The subject of the triple is the annotated element and should be formatted as a relative URI. The predicate is the URI of a BioModels.net qualifier linking the subject to a URI from a knowledge resource or the Dublin Core Metadata Terms (https://dublincore.org/specifications/dublin-core/dcmi-terms/) qualifier `description`. The object of the triple should be an identifiers.org-formatted URI indicating a concept in a knowledge resource, or a text string for free-text definitions of model elements.

The following is an example of a singular, model-level, non-semantic annotation that indicates the PubMed ID of the model's source publication. In this case, the metadata ID for the model as a whole (e.g., the metadata ID associated with the <model> element in an SBML model) is "modelmeta1":

```
<rdf:Description rdf:about="./MyModel.xml#modelmeta1">
 <bqmodel:isDescribedBy rdf:resource="https://identifiers.org/pubmed/12991237" />
</rdf:Description>
```

The following is an example singular semantic annotation indicating that the model element with metadata ID "meta1" from the model file "MyModel.xml" represents adenosine tri-phosphate:

```
<rdf:Description rdf:about="./MyModel.xml#meta1">
 <bqbiol:is rdf:resource="https://identifiers.org/chebi/CHEBI:15422" />
</rdf:Description>
```

The following is an example free-text description of a model variable with metadata ID "meta2":

```
<rdf:Description rdf:about="./MyModel.xml#meta2">
 <dcterms:description>Cardiomyocyte cytosolic ATP concentration</dcterms:description>
</rdf:Description>
```

#### 2.3.6 Serializing composite semantic annotations

Composite semantic annotations (CSAs) are used to capture the biological meaning of model or data elements when no singular reference term is available that provides a precise enough definition. Based on the SemSim framework [12], CSAs are comprised of two components: the *physical property* that is represented, and what it is a property *of*. The second component, the bearer of the property, is either a physical entity, process, force or dependency.

##### 2.3.6.1 *Composite annotation for a property of a physical entity*

Consider a CellML variable that simulates blood volume in the left coronary artery. The physical property simulated is volume; more precisely, *fluid* volume. This fluid volume is a property of blood in the lumen of the left coronary artery. Because there is no existing knowledge resource term that represents "blood volume in the left coronary artery", we instead construct a CSA using a combination of existing knowledge resource terms. For the physical property components of CSAs, we recommend using terms from the Ontology of Physics for Biology (OPB [13]) because the OPB provides a comprehensive, formally-structured hierarchy of physical properties. In this case, we would use the OPB term "Fluid volume" (OPB:OPB_00154). The second part of the CSA (blood in the left ventricle) can be created by linking two terms from the Foundational Model of Anatomy (FMA [14]), namely "Portion of blood" (FMA:9670) and "Lumen of left coronary artery" (FMA:18228). We link these two FMA terms using the **isPartOf** BioModels.net qualifier to produce a composite physical entity. The additional statements required in a CSA link the model element being annotated to the physical property it represents (via the **isVersionOf** BioModels.net qualifier) as well as to the composite physical entity that bears the property (via the **isPropertyOf** BioModels.net qualifier).

Encoded in RDF/XML, this CSA example would be serialized as:

```
<rdf:Description rdf:about="./MyModel.xml#VLV">
  <bqbiol:isVersionOf rdf:resource="https://identifiers.org/opb/OPB_00154" />
  <bqbiol:isPropertyOf rdf:resource="#entity_0"/>
</rdf:Description>

<rdf:Description rdf:about="#entity_0">
  <bqbiol:is rdf:resource="https://identifiers.org/fma/FMA:9670" />
  <bqbiol:isPartOf rdf:resource="https://identifiers.org/fma/FMA:18228"/>
</rdf:Description>
```

Figure 1 shows a node and edge diagram for the example CSA:

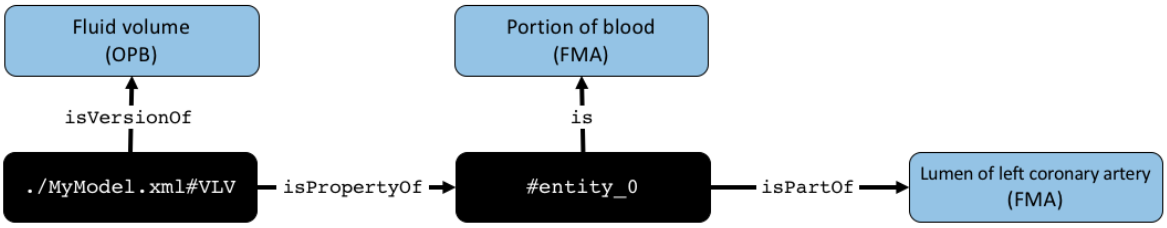

Figure 1. Node and edge representation of an example composite annotation for a model variable that simulates blood volume in the left coronary artery. RDF resources local to the OMEX metadata file are in black boxes, external knowledge resource terms are in blue boxes.

Note that the generic RDF resource `entity_0` needs to be created when encoding the CSA. This is required because there are no structures in the CellML schema that represent physical entities, so they are instantiated as RDF resources in the OMEX metadata document. Such instantiation is often not needed for SBML models, since SBML provides explicit XML elements for representing the bearers of physical properties including chemical species, compartments and reactions, and these elements can have unique metadata identifiers assigned to them.

However, instantiation of a resource that represents the full concept described by the CSA *is* needed for the properties of species, compartments, and reactions in SBML models. While SBML uses XML structures for declaring physical entities (<compartment> and <species> elements) as well as processes (<reaction> elements), it does not declare XML elements that represent the *properties* of those entities and processes. Instead, the property is indicated by an XML attribute on the entities and processes. Therefore, to ensure that these properties are searchable in OMEX annotations, we recommend instantiating a generic RDF resource for each physical property that is implicitly represented in an SBML model. Note that this does not have to be done when annotating an SBML parameter with a CSA because the SBML parameter element includes a metadata ID that can be explicitly referenced in the OMEX metadata file.

##### 2.3.6.2 Composite annotation for a property of a physical process

The example semantic annotation above is for a physical property of a physical entity, however, the physical properties simulated in models or measured experimentally can also be those of physical processes such as chemical reactions, transport of solutes, flow of fluids in vessels, etc. For CSAs on process properties, we recommend the approach created by the developers of the SemSim architecture wherein a custom physical process is instantiated and linked to its energetic sources and sinks as well as mediators whose amounts modulate the magnitude of the physical process property.

Sources, sinks and mediators are the physical entities that participate in the process: source amounts are consumed by the process, sink amounts are produced, and mediator amounts remain unchanged. Below, we provide an example of a CSA for the rate of a chemical reaction with one source, one sink, and one mediator.

First, we assert statements indicating that the model represents a physical property of a process that is a chemical flow rate (OPB:OPB_00592).

```
<rdf:Description rdf:about="./MyModel.xml#property_metaid_0">
  <bqbiol:isPropertyOf rdf:resource="./MyModel.xml#process_metaid_0"/>
  <bqbiol:isVersionOf rdf:resource="https://identifiers.org/opb/OPB_00592"/>
</rdf:Description>
```

If this annotation was on a CellML model containing a variable representing the chemical flow rate, the fragment `property_metaid_0` should point to the CellML variable with that metadata ID. If this annotation was on an SBML model that represents the flow rate using a <reaction> element and associated attributes, then `property_metaid_0` should be a physical property resource instantiated within the RDF content. The reason is that asserting chemical flow rates via the SBML <reaction> structure does not include the explicit construction of an XML element representing the flow rate that can be referenced in the OMEX metadata RDF.

We next assert statements that indicate the physical entity participants in the process: the energetic sources, sinks, and mediators. In this case, there is one source, one sink, and one mediator. The sources and sinks can include stoichiometry statements (using the `semsim:hasMultiplier` qualifier) to indicate the production/consumption ratios for the process participants (mediator participation statements do not include stoichiometries).

```
<rdf:Description rdf:about="./MyModel.xml#process_metaid_0">
  <semsim:hasSourceParticipant rdf:resource="./MyModel.xml#source_0"/>
  <semsim:hasSinkParticipant rdf:resource="./MyModel.xml#sink_0"/>
  <semsim:hasMediatorParticipant rdf:resource="./MyModel.xml#mediator_0"/>
</rdf:Description>

<rdf:Description rdf:about="./MyModel.xml#source_0">
  <semsim:hasMultiplier>1.0</semsim:hasMultiplier>
  <semsim:hasPhysicalEntityReference rdf:resource="./MyModel.xml#species_metaid_0"/>
</rdf:Description>

<rdf:Description rdf:about="./MyModel.xml#sink_0">
  <semsim:hasMultiplier>2.0</semsim:hasMultiplier>
  <semsim:hasPhysicalEntityReference rdf:resource="./MyModel.xml#species_metaid_1"/>
</rdf:Description>
```

```
<rdf:Description rdf:about="./MyModel.xml#mediator_0">
  <semsim:hasPhysicalEntityReference rdf:resource="./MyModel.xml#species_metaid_2"/>
</rdf:Description>
```

RDF statements indicating the biological identity of the chemical species that participate in the process (the resources with the `species_metaid_*` URI fragments in this example) would be included elsewhere in the RDF. For SBML models where these chemical species are explicitly represented using <species> elements, the metadata IDs should point to those elements in the SBML code. For other formats, such as CellML, that do not support such elements, the metadata IDs should point to physical entities instantiated elsewhere in the RDF.

We recognize that creating CSAs for biological processes in this manner can duplicate information that is present in an SBML model's reaction network structure. However, we have decided that CSAs for physical processes should be written out in accordance with these guidelines so that all biological features represented in a model are exposed in the RDF metadata alone. This way, the community can more easily apply RDF-processing tools to analyze, query, and reason over semantic metadata in COMBINE archives.

##### 2.3.6.3 Composite annotation for a property of a physical force

CSAs can also be used to represent the properties of physical forces. These include, for example, membrane potentials, chemical potentials and fluid pressures. The structure of CSAs on force properties is similar to process properties. Because forces themselves are not conventionally named or represented explicitly in model code, a force is instantiated as a local resource in the RDF with its energetic sources and sinks specified. (Mediators are only used for process property annotations and stoichiometries are undefined for force participation statements.)

The following is an example CSA that represents the electrical potential caused by a difference in the amount of charged ions on either side of a cell membrane. For this example, we assume a model element with metadata ID "parameter_metaid_0" represents this biological property.

We assert the triples stating that the model element represents a property of a force, and that it represents the electrical potential (OPB:OPB_01058) of that force:

```
<rdf:Description rdf:about="./MyModel.sbml#parameter_metaid_0">
  <bqbiol:isPropertyOf rdf:resource="./MyModel.sbml#force_0"/>
  <bqbiol:isVersionOf rdf:resource="https://identifiers.org/opb/OPB_01058"/>
</rdf:Description>
```

We add triples that indicate the physical entity participants (energetic sources and sinks) whose properties establish the force:

```
<rdf:Description rdf:about="./MyModel.sbml#force_0">
  <semsim:hasSourceParticipant rdf:resource="./MyModel.sbml#source_0"/>
  <semsim:hasSinkParticipant rdf:resource="./MyModel.sbml#sink_0"/>
</rdf:Description>

<rdf:Description rdf:about="./MyModel.sbml#source_0">
  <semsim:hasPhysicalEntityReference rdf:resource="./MyModel.sbml#species_metaid_0"/>
</rdf:Description>

<rdf:Description rdf:about="./MyModel.sbml#sink_0">
  <semsim:hasPhysicalEntityReference rdf:resource="./MyModel.sbml#species_metaid_1"/>
</rdf:Description>
```

For SBML models, the URI fragments `species_metaid_0` and `species_metaid_1` would correspond to metadata IDs on <species> elements in the SBML code. Importantly, for models in CellML or other formats that do not include the explicit representation of physical entities, these metadata IDs would point to physical entity resources instantiated elsewhere in the RDF metadata.

##### 2.3.6.4 *Composite annotation for a property of a physical dependency*

The final type of physical properties that are represented in simulation models are properties of physical dependencies. Also known as “constitutive properties”, these include, for example, electrical resistance, fluid volumetric elastance, and reaction rate constants. Unlike entity, process, and force properties, dependency properties characterize the mathematical relationship between *two or more* physical properties. For example, linear electrical resistance is defined as the slope of the relationship relating electrical potential across a resistor and the electrical current through it.

Currently, we advise annotators to only indicate the represented physical property for model elements that quantify these types of properties. We believe it is sufficient to say that a certain model parameter or variable “is an electrical resistance” rather than encode all information about the physical properties that play a role in the dependency. To determine which specific physical properties in the model are related through the dependency (and thus, which physical entities, processes and/or forces), software libraries should be able to examine the equation solving for the constitutive property and then identify which physical role players are involved in the dependency based on the identifiers used in the equation.

#### 2.3.7 Annotating tabular data

Biomedical data is often stored and shared in plain-text, delimited tables organized into rows and columns. Annotating these tables using OMEX metadata would provide a way to describe the data in more detail than the plain-text format provides. Therefore, we recommend that OMEX metadata libraries provide basic support for serializing and retrieving annotations on tabular data files within COMBINE archives. While more sophisticated strategies may emerge with time, for now we recommend that libraries support annotating individual columns within tabular data files using the column header as a surrogate metadata ID that is referenced in annotation statements within the OMEX metadata file. For example, if a data file contains a column with values that represent the volume of blood in the left coronary artery recorded over time, the meaning of the data in the column could be captured using the CSA example in section 2.3.6.1. The composite annotation would look the same as in the example, except the URI for the subject of the first statement would refer to a data file and a column header (`./MyData.csv#VleftCorArt` in the RDF below):

```
<rdf:Description rdf:about="./MyData.csv#VleftCorArt">
  <bqbiol:isVersionOf rdf:resource="https://identifiers.org/opb/OPB_00154" />
  <bqbiol:isPropertyOf rdf:resource="#entity_0"/>
</rdf:Description>

<rdf:Description rdf:about="#entity_0">
  <bqbiol:is rdf:resource="https://identifiers.org/fma/FMA:9670" />
  <bqbiol:isPartOf rdf:resource="https://identifiers.org/fma/FMA:18228"/>
</rdf:Description>
```

Note that this strategy for annotating data requires column headers to be unique within a data file. We encourage the community to continue to develop strategies for annotating data in commonly-used formats.

#### 2.3.8 Annotating physical units

Currently, the COMBINE community does not have a consensus, cross-format approach for annotating the meaning of physical units declared in models or for indicating the physical units on experimental data values. We recommend that the community develop strategies to support physical unit annotation so that software tools can easily recognize unit mismatches when comparing semantically equivalent model elements (for example, during model merging) or when associating model variables/parameters with experimental data.

### 2.4 Knowledge resources to use for annotation

Different members of the modeling community may prefer to use different ontologies or databases when annotating their models and associated files. Therefore, software packages that adhere to this specification should allow annotators to use a wide variety of knowledge resource terms. However, in the interest of introducing a degree of standardization to the annotation process, we provide the following list of recommended knowledge resources. Note that this list primarily focuses on annotation of models and does not address other file types (SED-ML, for example) that may be packaged in COMBINE archives.

#### 2.4.1 Resources to use for model-level annotations

The specific knowledge resources used for model-level annotations largely depend on the curatorial objectives of model development teams. Thus, we cannot make comprehensive recommendations about which resources should be used for these annotation types. However, to link a model to its source publication, we recommend using the publication's PubMed ID or DOI. To indicate the taxon for which the model is applicable, we recommend using identifiers from the NCBI taxonomy resource. To indicate the physical bounds within which a model's simulated phenomena occur, we recommend applying a singular model-level annotation that uses the `bqbiol:occursIn` relation to link the model to a term from, for example, one of the physical entity knowledge resources listed in the following section.

#### 2.4.2 Resources to use for composite semantic annotations

The SemSim development group makes the following recommendations for which knowledge resources to use when creating a composite semantic annotation:

**Physical *property* component of a CSA**

- Ontology of Physics for Biology (OPB)

**Physical *entity* component of a CSA**

- Chemical Entities of Biological Interest (ChEBI) for atoms and small molecules (e.g., metabolites)
- Protein Ontology (PR) for proteins
- UniProt can also be used to annotate proteins in a model when it is important to disambiguate the proteins based on their amino acid sequence or taxonomic species
- Gene Ontology:cellular component for subcellular structures
- Cell Type Ontology (CL) for cell types
- Foundational Model of Anatomy (FMA) for structures at the tissue scale or higher
- Mouse Adult Gross Anatomy (MA) ontology for rodent-specific gross anatomy
- Ontology for Biomedical Investigations (OBI) for laboratory materials

**Physical *process* component of a CSA**

Physical processes are always defined using a custom (*ad hoc*) term; however, `bqbiol:isVersionOf`, `bqbiol:isPartOf` and `bqbiol:hasPart` statements can be added to the custom term to give it semantic context. The recommended knowledge resources to use for those statements include:

- Gene Ontology:biological process

#### 2.4.3 Resources to use for singular physical property annotations

In cases where the CSA structure is insufficient for capturing the meaning of a model element, singular terms from knowledge resources may suffice. However, more research on the annotation needs of the biosimulation community as well as the limitations of the CSA approach is needed before recommendations can be made regarding which resources to use for this purpose.

#### 2.4.4 Internally referencing biological knowledge stored within COMBINE archives

Depending on their research domain, some modelers may find that external knowledge resource terms that sufficiently disambiguate elements in their model are unavailable. In such instances we recommend making term requests to maintainers of the appropriate knowledge resources so that reference terms needed by the community are added to those resources. We also recommend that software packages supporting this specification provide functions and services to expedite these requests.

However, for researchers in some modeling domains, the annotation terms needed to disambiguate model elements may require a level of detail not supported among current biomedical knowledge resources. Thus, some researchers may need to compose complete descriptions of their own knowledge resource terms to define a model element. Since these descriptions may not be aggregated into publicly-available collections, and thus may not possess web-resolvable URIs, they should be storeable within COMBINE archives so they can be referenced in OMEX annotation statements.

For example, this issue arises in modeling protein modifications such as phosphorylation. A complete collection of knowledge resource terms that circumscribe all possible protein phosphorylations is unavailable, and may be untenable in terms of maintenance. This issue may also arise in synthetic biology models where model elements are disambiguated by nucleotide or amino acid sequences, and where non-canonical biopolymers are represented.

We therefore recommend that OMEX annotation software packages support annotation statements that link an annotated item to an *ad hoc* knowledge term stored within the COMBINE archive. For example, an annotator should be able to reference a nucleotide or amino acid sequence in a FASTA file contained in the archive in order to link, say, a non-canonical protein in an SBML model to the sequence description that defines it. Any encoded knowledge items stored within the OMEX file should include a unique identifier (e.g., those used in the headers of FASTA file enties) so that they can be used in RDF annotation statements linking models and/or data elements to knowledge items within the archive.

RDF statements that reference an internal knowledge term should do so using a URI composed of the path to the file in the archive followed by the unique identifier for the item. For example, the URI for an entry with the unique identifier “seq1” in a top-level FASTA file named “MySeqs.fasta” should be

```
./MySeqs.fasta#seq1
```

OMEX annotation software libraries should interpret the fragment in these URIs as an entry in the unique identifier field of whatever format the knowledge item is stored in. For a FASTA file, the fragment would indicate the text in the header/identifier field of a FASTA entry. Other formats for storing knowledge terms might include the Synthetic Biology Open Language (SBOL [15]) , BioPax [16], BpForms [17], BcForms [17], the Web Ontology Language (OWL, `https://www.w3.org/TR/owl2-overview/`, or the Open Biomedical Ontology (OBO) format [18].

### 2.5 OMEX Metadata resources

Community discussions on OMEX metadata issues can be found at the combine-annot Google group: `https://groups.google.com/forum/#!forum/combine-annot`.

A C/C++ library to support this specification is currently under development at `https://github.com/sys-bio/libsemsim`

Examples of OMEX metadata annotations can be found at the combine-org/Annotations GitHub site: `https://github.com/combine-org/Annotations`

Information on the COMBINE archive format can be found at `http://co.mbine.org/standards/omex`

## 3 Acknowledgements

This specification has been developed with the valuable input of Daniel Cook, Jonathan Cooper, Andreas Dräger, Alan Garny, Nick Juty, Jonathan Karr, Goksel Misirli, Chris Myers, and Herbert Sauro.

## A OMEX Metadata examples

Examples of annotated models that adhere to this specification are available at `https://github.com/combine-org/Annotations/tree/master/standardized`

## B The COMBINE archive

A COMBINE archive is a single file containing the various documents (and in the future, references to documents), necessary for the description of a model and all associated data and procedures. This includes for instance, but is not limited to, simulation experiment descriptions in SED-ML, all models needed to run the simulations in SBML and their graphical representations in SBGN-ML. It is a convenient alternative if a model source URI cannot be resolved, or if an end-user is offline.

The SED-ML archive described in appendix D of the SED-ML Level 1 Version 1 specification formed the basis for the COMBINE archive with contributions from the SED-ML and COMBINE communities.

The COMBINE archive is described at: `https://co.mbine.org/documents/archive`.
